# Effects of Temperature, pH, and Relative Humidity on Growth of *Penicillium crustosum* OM1 Isolated from Pears and Its Penitrem A Production

**DOI:** 10.3390/jof11100741

**Published:** 2025-10-16

**Authors:** Shengming Gao, Sung-Yong Hong, Gyu-Mi Jung, Ae-Son Om

**Affiliations:** Department of Food and Nutrition, College of Human Ecology, Hanyang University, Seoul 04763, Republic of Korea; gaosm97@gmail.com (S.G.); lunohong@hanyang.ac.kr (S.-Y.H.); mb3159@hanyang.ac.kr (G.-M.J.)

**Keywords:** penitrem A, *Penicillium crustosum* OM1, pH, relative humidity, temperature

## Abstract

Penitrem A, an indole–diterpenoid neurotoxin, is produced by several species of *Penicillium* on cereal grains, meat, dairy products, and fruits. *Penicillium crustosum* is a well-known penitrem A producer, but much is unknown about physiological characteristics of *P. crustosum*. In this study, we isolated penitrem A-producing *P. crustosum* OM1 from pears, and investigated the influence of temperature, pH, and relative humidity [RH] on its growth and penitrem A production. The fungal species exhibited the highest growth at 25 °C and pH 4.5 on mYES4 under RH 98%, whereas it produced the highest level of penitrem A at 22 °C and pH 6.5 on the same media under RH 98%. Furthermore, RT-qPCR analysis of six penitrem A biosynthetic genes (*ptmB*, *ptmJ*, *ptmK*, *ptmO*, *ptmS*, and *ptmT*) expression in *P. crustosum* OM1 showed that the four penitrem A biosynthetic genes (*ptmJ*, *ptmK*, *ptmO*, and *ptmS*) were up-regulated in mYES4 (penitrem A conducive medium), whereas they were not in mMEB (penitrem A non-conducive medium). Our results demonstrated that the three key environmental factors significantly affected the growth of *P. crustosum* OM1 and its penitrem A production. These findings could help find efficient methods to prevent penitrem A contamination from fresh fruits including pears.

## 1. Introduction

Mycotoxins are toxic metabolites produced by various filamentous fungi such as *Penicillium* spp. and *Aspergillus* spp. on agricultural commodities such as cereal grains, fruits, and vegetables during pre-harvest and post-harvest storage. To date, more than 300 different mycotoxins have been reported [1]. As most mycotoxins are heat resistant, it is difficult to remove them from food materials during food processing. Thus, when consumed, they can cause diverse toxic health effects in several target organs such as liver, kidney, and immune or nervous systems in humans and animals [2].

Of the mycotoxins, penitrems are one group of emerging mycotoxins, which belong to indole diterpenes, found in food products including moldy cream cheese and chestnuts [3,4]. Penitrem A is a well-studied neurotoxin produced by certain species of *Penicillium* among seven penitrems (A–G) [5,6]. It has been reported that it was involved in intoxication in humans and animals after ingestion of food products including moldy cheese, walnuts, bread, rice, and canned soup [7,8,9,10,11]. It can cause vomiting, mydriasis, polypnea, ataxia, tremors, convulsion, and muscle incoordination [4,11]. The penitrem A toxicity is believed to result from the increased release of neurotransmitters, including ϒ-aminobutyric acid (GABA), glutamate, and aspartate, from brain cells, and/or inhibition of Ca^2+^-activated K^+^ channels in smooth muscle cells [12,13,14,15]. Recently, several previous studies established the penitrem A biosynthetic pathway [16,17,18]. Penitrem A is produced via complex reactions starting with indole-3-glycerol phosphate by 20 biosynthetic genes in the penitrem A biosynthetic gene cluster.

Another mycotoxin, patulin, is produced by some species of *Penicillium* and *Aspergillus* on mainly fruits including apples and pears [19]. Since patulin can cause several toxicities such as neurotoxicity, immunotoxicity, genotoxicity, and carcinogenicity in humans, it is classified as a Group 3 carcinogen by the International Agency for Research on Cancer (IARC) [19,20].

On the other hand, it has been documented that penitrem A is produced by certain species of *Penicillium* sp. such as *Penicillium crustosum*, *Penicillium glandicola*, *Penicillium flavigenum*, *Penicillium antarcticum*, and *Penicillium carneum* [17,21]. Of penitrem A-producing fungi, *P. crustosum* is ubiquitous in soil and known to be the most frequently found in food products and the main producer of the mycotoxin [5]. *P. crustosum* has been primarily isolated from meat, cereal grains, nuts, dairy products, fruits, and vegetables [3,22,23,24,25]. Some strains of them were not able to produce penitrem A on synthetic media due to strain-dependent variability although *P. crustosum* isolates from fruits such as lemon, grape, and mangosteen produced the mycotoxin [3,22,24,26]. Additionally, several environmental factors such as temperature, pH, and relative humidity (RH) can influence both fungal growth and mycotoxin production on food products [27,28,29]. It has been known that different fungal species have different optimum temperatures and limit RH for their growth [27]. Also, a *Penicillium expansum* strain, a patulin-producing isolate, produced the highest level of patulin at 16 °C and pH 4.0, whereas a patulin-producing *Penicillium paneum* OM1 produced the highest amount of patulin at 20 °C and pH 4.5 [28,29]. In addition, the *P. expansum* strain was not able to produce patulin below RH 99%, while the *P. paneum* OM1 was able to produce patulin even under RH 92% [28,29]. Much is unknown about the environmental parameters for penitrem A production by *P. crustosum*. Thus, in the current study, we isolated penitrem A-producing *P. crustosum* OM1 from pears, and investigated the optimal temperature, pH, and RH conditions for its growth and penitrem A production after incubation under different culture conditions. Also, we evaluated the relative gene expression levels of six major penitrem A biosynthetic genes (*ptmB*, *ptmJ*, *ptmK*, *ptmO*, *ptmS*, and *ptmT*) in *P. crustosum* OM1 under penitrem A supportive and non-supportive synthetic media. Our findings in the present study could be utilized for development of efficient strategies to control fungal growth and penitrem A contamination on fresh fruits such as pears.

## 2. Materials and Methods

### 2.1. Chemicals and Reagents

Penitrem A standard (≥95.0% purity) was purchased from Enzo Life Sciences Inc. (Farmingdale, NY, USA), and patulin standard (≥98.0% purity), acetic acid (≥98.0%, crystalline), trifluoroacetic acid (≥99.0% purity), Tween 80, proteinase K, disodium ethylenediaminetetraacetic acid (Na_2_EDTA), and ethanol were obtained from Sigma-Aldrich Co., (St. Louis, MO, USA). Ethyl acetate was purchased from Daejung chemical Co., (Seoul, Republic of Korea), and sodium carbonate (Na_2_CO_3_) and methanol (≥99.99% purity) were from Samchun chemical Co., (Seoul, Republic of Korea). Acetonitrile (ACN; ≥98.0% purity) was obtained from J. T. Baker (Avantor Performance Materials Inc., Radnor, PA, USA), and sodium acetate, anhydrous sodium sulfate (Na_2_SO_4_), potassium sulfate (K_2_SO_4_), and potassium nitrate (KNO_3_) were from Junsei chemical Co., (Tokyo, Japan). Phenol/chloroform/isoamyl alcohol (25:24:1) was purchased from Biochemicals Inc. (Gyeonggi, Republic of Korea), and sodium dodecyl sulfate (SDS), tris base, and 2-mercaptoethanol were from Bio-Rad (Hercules, CA, USA).

### 2.2. Isolation of Fungal Strains from Pears

Fresh pears (cultivar Soojeong) were used to search for patulin- or penitrem A-producing fungal strains after harvest at Asan Agricultural Technology Service Center (ATST; Asan, Chungcheongnam, Republic of Korea) in the spring of 2023. After preparation of microbial cell suspension from surface of the collected pears by the cotton swab method as described previously [29], the suspension was inoculated onto potato dextrose agar (PDA; MB Cell, Seoul, Republic of Korea) plates containing tetracycline and chloramphenicol (1 mg of tetracycline and 1 mg of chloramphenicol in 200 mL of PDA) and incubated at 30 °C for 4 days. For isolation of pure fungal strains, each fungal isolate was transferred onto a new PDA plate and incubated at 30 °C for 4 days. After spore preparation with 0.01% Tween 80 solution, diluted spore suspension was inoculated onto new PDA plates, which was repeated three times.

### 2.3. Morphological and Genetic Identification of Fungal Isolates and Phylogenetic Analysis

For microscopic observation of morphological structures, 2 × 10^4^ fungal spores were center-inoculated onto each PDA or modified malt extract agar (mMEA; 2% malt extract, 2% dextrose, 0.1% peptone, 0.0005% CuSO_4_·7H_2_O) plate and incubated at 30 °C for 5 days after spore preparation with 0.01% Tween 80 solution. The morphological structure of each fungal isolate was then observed using a lactic acid slide mount under a microscope (Olympus CHK2-F-GS, Olympus Co., Ltd., Tokyo, Japan).

For molecular identification, fungal isolates were analyzed based on sequences of 3 DNA regions (internal transcribed spacer [ITS], β-tubulin [BenA], and calmodulin [CaM]) [30,31,32]. Briefly, approximately 10^7^ of fungal spores were inoculated into 100 mL of potato dextrose broth (PDB; MB Cell, Seoul, Republic of Korea) and incubated at 30 °C for 5 days with shaking at 150 rpm for genomic DNA isolation. Genomic DNA was extracted from fungal mycelia by a procedure of Steven B. Lee and John W. Taylor with minor modifications [33] as described previously [29]. Next, the 3 regions (ITS, BenA, and CaM) of the isolated genomic DNA were amplified by polymerase chain reaction (PCR) along with a set of 2 specific primers for each region to identify fungal species as described previously [30,31,32,34]. The primer sequences are as follows: ITS1 (5′-TCCGTAGGTGAACCTGCGG-3′, forward) and ITS4 (5′-TCCTCCGCTTATTGATATGC-3′, reverse) for ITS region, Bt2a (5′-GGTAACCAAATCGGTGCTGCTTTC-3′, forward) and Bt2b (5′-ACCCTCAGTGTAGTGACCCTTGGC-3′, reverse) for BenA region, and Cmd5 (5′-CCGAGTACAAGGAGGCCTTC-3′, forward) and Cmd6 (5′-CCGATAGAGGTCATAACGTGG-3′, reverse) for CaM region. After the PCR products were run on 1.2% (*w*/*v*) agarose gels by electrophoresis, they were purified using AccuPep PCR/Gel Purification Kit (Bioneer, Daejeon, Republic of Korea), and sequenced by Biofact Co., (Daejeon, Republic of Korea). The fungal isolates were then identified by the local similarity between DNA sequences of the PCR products and fungal strains, which were retrieved from GenBank in National Center for Biotechnology Information (NCBI).

The phylogenetic tree was constructed using the MEGA program (v. 11), based on the neighbor-joining (NJ) method, along with DNA sequences of identified fungal species [35].

### 2.4. Fungal Culture Conditions

Approximately 10^6^ fungal spores were inoculated into 5 mL of PDB and incubated at 30 °C and 120 rpm for 5 days to screen for patulin- or penitrem A-producing fungal strains after spore preparation with 0.01% Tween 80 solution as described above.

For fungal culture on modified yeast extract sucrose (mYES; 2% yeast extract, 4 or 15% sucrose, 0.1% peptone, 0.1% MgSO_4_·7H_2_O, 0.0005% CuSO_4_·7H_2_O) agar plates containing different sucrose concentrations, after mYES plates were covered with a sterilized cellophane disk (GelAir Cellophane Support; Bio-Rad, Hercules, CA, USA), 2 × 10^4^ spores were center-inoculated onto each plate and incubated at 22 °C for 16 days.

For fungal culture on PDA, mYES4 (4% sucrose), mMEA, and modified Czapek yeast agar (mCYA; 4.9% Czapek Dox, 0.5% yeast extract, 0.0005% CuSO_4_·7H_2_O) plates, fungal spores (2 × 10^4^) were center-inoculated onto each agar plate as described above. The agar plates were then incubated for 16 days under 5 different temperature conditions (15, 20, 22, 25, and 30 °C).

For fungal culture under 4 different pH conditions (4.5, 6.5, 7.5, and 8.5), mYES4 plates were prepared by adjustment of the media pH (pH 6.8, unadjusted pH) using HCl or NaOH. The mYES4 plates were then incubated at 22 °C for 16 days after inoculation as described above. Throughout this study, 0.0005% CuSO_4_·7H_2_O was added to prepare mYES4, mMEA, and mCYA according to a report on its stimulation of penitrem A production by *P. crustosum* CBS 483.75 [5].

For fungal culture on pear puree agar medium (PPAM), after preparation of PPAM plates (pH 6.5) containing homogenized pears (cultivar Soojeong) by adjustment of the media pH (pH 5.0, unadjusted pH), 2 × 10^4^ spores were center-inoculated onto each PPAM plate as described above. The PPAM plates were then incubated at 22 °C for 16 days. For fungal culture under different RH conditions, after RH of 4 containers was adjusted to approximately 94 or 98% using saturated salt solutions (KNO_3_ for 94% RH and K_2_SO_4_ for 98% RH at 22 °C) [36,37], PPAM (pH 6.5) and mYES4 (pH 6.5) plates were placed in the covered containers (20 cm × 15 cm × 8 cm; Daiso, Seoul, Republic of Korea; 6 PPAM plates in one container) and incubated at 22 °C for 10 days.

For microscopic observation of morphological structures, fungal spores (2 × 10^4^) were center-inoculated onto each PDA, PPAM, mMEA, mYES4, and mCYA plate and incubated as described above. The morphological structures were then observed using lactic acid slide mounts under a microscope (Olympus IX 71, Olympus Co., Ltd., Tokyo, Japan).

For liquid culture, fungal spores (10^6^) were inoculated into 100 mL of mYES4 (pH 6.8), mMEB (pH 6.4), or PDB (pH 5.0), and incubated at 22 °C for 8 days under static or agitation conditions at 160 rpm.

For relative expression analysis of penitrem A biosynthetic genes by RT-qPCR, fungal spores (10^6^) were inoculated into 100 mL of mYES4 or mMEB liquid medium and incubated at 22 °C for 9 days under static (for mYES4) or agitation (for mMEB) conditions at 160 rpm.

### 2.5. Measurement of Fungal Growth Rates

Fungal growth rates were assessed by measuring the dry weight of mycelia and the diameter of colonies grown on PPAM and mYES plates. The diameter of the colony was analyzed by measuring the 2 diameters of the colony at right angles to each other [28]. Mycelial dry weight was determined after the mycelia grown on PPAM and mYES were completely dried at 90° C. It was also measured with colonies grown on mMEA, mCYA, and PDA plates, and whole mycelia grown in mYES4, PDB, and mMEB.

### 2.6. Patulin or Penitrem A Standard Solution

Patulin standard solutions for high performance liquid chromatography (HPLC) analysis were prepared as described previously [29].

A penitrem A stock solution (1 mg/mL) was prepared by dissolving 1 mg of penitrem A powder in 1 mL of methanol and stored at −20 °C. A series of penitrem A standard solutions (0.1, 0.5, 1.0, 2.5, 5.0, and 10.0 μg/mL) were prepared freshly by dilutions of the stock solution with methanol. Then, after 1 mL of each standard solution was evaporated to dryness under nitrogen at 45 °C, the residue was dissolved in 1 mL of 70% ACN (ACN:DW = 70:30, *v*/*v*).

### 2.7. Patulin or Penitrem A Extraction

Patulin extraction was performed using the AOAC official method 995.10 with minor modifications [38] as described previously [29].

Penitrem A was extracted according to Moldes-Anaya and collaborators’ method with slight modifications [39]. Briefly, for penitrem A extraction from liquid or solid culture, each culture was extracted twice with 90% ACN (ACN:DW = 90:10, *v*/*v*) by shaking for 1 h using a Wrist Action Shaker (Burrell Scientific, Pittsburgh, PA, USA); for penitrem A extraction from liquid culture, after each culture was filtered through a filter paper [Whatman, Maidstone, UK], the filtered mycelium was used for the extraction because previous studies showed that penitrem A is an intracellular metabolite and accumulates within fungal mycelia [97–99%] rather than liquid culture media [22,40,41]. The extract was then filtered through a filter paper (GF/A; Whatman, Maidstone, UK). After it was dried under nitrogen at 45 °C. The residue was dissolved in 70% ACN (ACN:DW = 70:30, *v*/*v*) and filtered through a 0.2 μm polyvinylidone fluoride (PVDF) syringe filter (Hyundai Micro Co., Seoul, Republic of Korea).

### 2.8. HPLC Analysis

Patulin was analyzed using an HPLC system (LC-20AT, Shimadzu; Tokyo, Japan) equipped with UV detector (UVD; SPD-10A, Shimadzu; Tokyo, Japan) system as described previously [29].

Penitrem A was quantified through a ZORBAX Eclips plus C18 column (4.6 mm × 250 mm, 5 μm particle size, Agilent; Santa Clara, CA, USA) by the above same HPLC-UVD system at 233 nm. The column oven temperature was set at 40 °C, and the injection volume of samples was 10 μL. The mobile phase consisted of solvent A (DW containing 0.05% trifluoroacetic acid [TFA]) and solvent B (ACN containing 0.05% TFA), and was pumped into the HPLC system at a constant flow rate of 4 mL/min. A gradient elution program was applied as follows: after B solution rapidly increased to 5% from 0 min to 0.01 min, it was held on 5% from 0.01 min to 5 min. Then, it linearly increased from 5% at 5 min to 100% at 25 min. After it was maintained on 100% from 25 min to 30 min, the 100% B solution rapidly decreased to 5% at 31 min. The 5% B solution was maintained for 2 min for re-equilibration of the column, giving a total run time of 33 min.

The linearity of a series of patulin or penitrem A concentrations in the HPLC analysis was evaluated by a calibration curve using 5 levels of patulin or 6 levels of penitrem A standard solutions (0.1, 0.2, 0.5, 1.0, and 2.0 μg/mL for patulin; 0.1, 0.5, 1.0, 2.5, 5.0, and 10.0 μg/mL for penitrem A). The calibration curve for each toxin was constructed by plotting the peak areas (y axis) versus concentrations of each toxin (x axis) in the HPLC-UVD analysis. The linearity was assessed by linear regression analysis and expressed as a coefficient of determination (r^2^). The r^2^ value of the patulin calibration curve was 0.999, while that of the penitrem A calibration curve was 0.998 (Figure S1).

The sensitivity of the HPLC-UVD method was determined by a limit of detection (LOD) and limit of quantification (LOQ). They were calculated using the slope (S) of the calibration curve and the standard deviation (SD) of the response, which were obtained from the above linearity assessment, as follows:

$$LOD=\frac{3.3\times SD}{S}$$

$$LOD=\frac{10\times SD}{S}$$

The LOD and LOQ for patulin were 0.006 and 0.018 μg/mL, whereas those for penitrem A were 0.005 and 0.014 μg/mL, respectively.

### 2.9. LC/MS/MS Analysis

The identity of penitrem A, which was extracted from liquid or solid culture, was confirmed by liquid chromatography–tandem mass spectrometry (LC/MS/MS). It was performed using an Agilent 1290 Infinity UHPLC system (Santa Clara, CA, USA), which was coupled to an Agilent 6545XT LC/Quadrupole Time-of-Flight (Q-TOF) LC/MS (Santa Clara, CA, USA) equipped with a dual-spray Agilent Jet Stream electrospray ionization source. Separation of analytes was carried out on an Agilent ZORBAX Eclipse Plus C18 column (100 mm × 2.1 mm, 1.8 μm particle size) (Santa Clara, CA, USA). The mobile phase consisted of solvent A (100% pure water containing 0.1% formic acid) and solvent B (100% methanol) with a flow rate of 0.25 mL/min. The column oven temperature was set at 40 °C and the injection volume of samples was 5 μL. A gradient elution program was applied as follows: after B solution increased to 5% from 0 min to 5 min, it continuously increased from 5% at 5 min to 95% at 15 min. Then, it was maintained on 95% from 15 min to 17 min. Subsequently, the 95% B solution decreased rapidly from 95% at 17 min to 5% at 18 min, and the 5% B solution was maintained for 1 min for re-equilibration of the column, giving a total run time of 19 min.

The general MS parameters in the positive mode were optimized and finally set as follows: capillary voltage in the positive mode, 3500 V; nozzle voltage in the positive mode, 300 V; drying gas temperature, 325 °C; drying gas flow rate, 6 L/min; nebulizer gas pressure, 45 psi; sheath gas temperature, 350 °C; sheath gas flow rate, 11 L/min. The peak spectrum was acquired using the Find by Formula data-mining algorithm. Agilent MassHunter Qualitative Analysis Software (rev. 10.0; Santa Clara, CA, USA) was used to process data.

### 2.10. Relative Gene Expression Analysis by RT-qPCR

After the fungal strain was cultured in mYES4 without shaking or mMEB with shaking and harvested as described above, the mycelia were promptly frozen in liquid nitrogen to extract total RNA. In addition, after penitrem A was extracted from mycelia, the levels of penitrem A in the extract were quantified by HPLC analysis as described above.

Total RNA extraction was performed with mycelia using RNeasy Mini Kit (QIAGEN, Hilden, Germany) following an instruction provided by the manufacturer. The RNA quality was assessed by Agilent 2100 Bioanalyzer (Agilent Technologies, Santa Clara, CA, USA).

Specific primer sets of penitrem A biosynthetic genes were designed using Primer3Plus on the basis of the genome sequence of *P. crustosum* OM1 (Table 1) [42].

The specificity of the primer sequences was evaluated using the Primer-BLAST at NCBI (https://www.ncbi.nlm.nih.gov/tools/primer-blast/index.cgi; 9 May 2024) [43,44] The β-tubulin gene of *P. crustosum* IMI 091917 (GenBank accession number AF003538.1) was used to design a set of specific primer sequences for an internal control [45].

The cDNA synthesis was performed using PrimerScript RT Reagent Kit with gDNA Eraser (Perfect Real Time) from TaKaRa (Takara Bio Inc., Shiga, Japan) according to the manufacturer’s instructions as described previously [29].

For RT-qPCR amplification, TB Green Premix Ex Taq II (Tli RNaseH Plus) ROX plus Kit (Takara Bio Inc., Shiga, Japan) was used, and the reactions were conducted following a procedure provided by the manufacturer as described previously [29]. RT-qPCR reactions were carried out at 95 °C for 5 min (an initial denaturation step), which was followed by 40 cycles of denaturation at 95 °C for 10 s, annealing at 58 °C for 30 s, and extension at 72 °C for 5 s [45]. After the final amplification cycle, a dissociation curve analysis of the PCR products was performed by a 0.5 °C increase per cycle from 60 to 95 °C to verify formation of a single PCR product. The data were analyzed using CFX Maestro software (v. 4.1; Bio-Rad, Hercules, CA, USA) and the relative gene expression level was calculated by the 2^−ΔΔCT^ method (comparative CT method) [46]. The β-tubulin gene was used as an internal control for normalization. The RT-qPCR analysis was performed with 3 independent biological samples, and 2 replicates were analyzed for each biological sample.

### 2.11. Statistical Analysis

Statistical analyses were performed by a one-way analysis of variance (ANOVA), which was followed by Duncan’s post hoc test. Data were expressed as the mean ± SD using SPSS 26.0 (SPSS Inc., Chicago, IL, USA). A *p* value < 0.05 was considered statistically different.

## 3. Results

### 3.1. Isolation of Fungi from Pears, Identification of the Isolated Fungi, and Penitrem A Production by the Isolated Fungi

**Figure 1:**
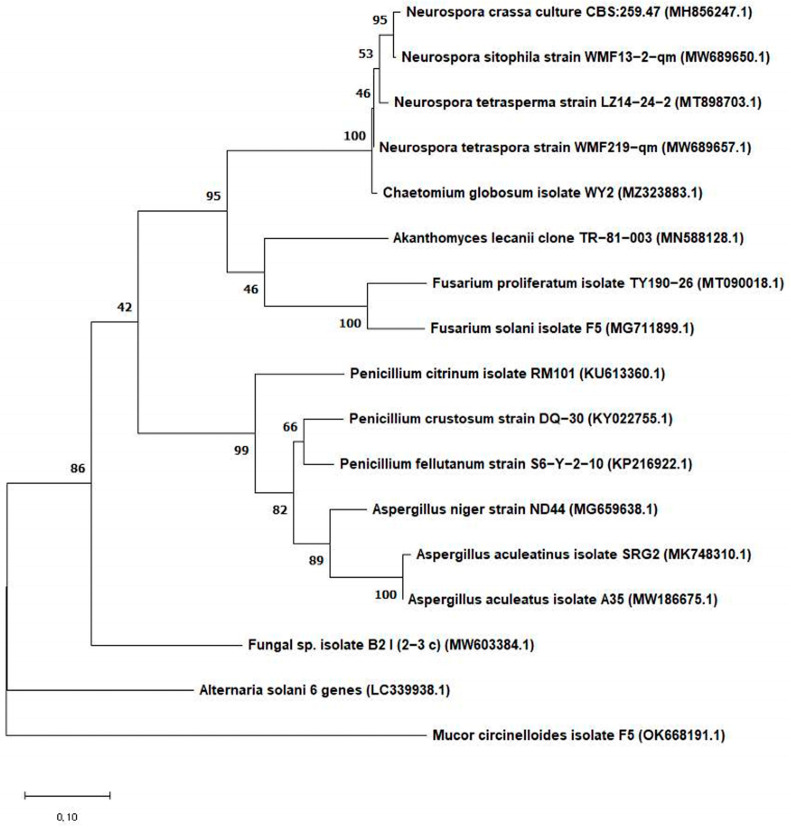
Phylogenetic relationship based on sequences of the ITS rDNA region from fungi isolated from pears. The trees were constructed using the neighbor-joining (NJ) method.

In total, 381 fungi were isolated from the surface of five pears. The fungal isolates were initially assigned to 16 groups based on their morphological characteristics such as conidiophores and conidiospores under the microscope, and the color and shape of the colony grown on PDA, mYES4, and mMEA agar plates. Then, we selected 17 fungal isolates from the 16 groups, and they were identified genetically based on sequences of ITS, BenA, and CaM regions on fungal DNA. When the sequences in the PCR products were compared with those of reference strains in the GenBank database at the NCBI website using Basic Local Alignment Search Tool (BLAST), the BLAST-based analysis showed high sequence similarity (98–100%) between the nucleotide sequences of the fungal isolates and those of the reference strains. The phylogenetic tree based on ITS sequences from 17 fungal isolates is shown in Figure 1. This result implies that pears were colonized by various fungi.

The 17 fungal isolates were classified into eight genera except one unknown genus (Fungal sp. isolate B21): *Neurospora* (four species), *Penicillium* (three species), *Aspergillus* (three species), *Fusarium* (two species), *Chaetomium* (one species), *Alternaria* (one species), *Mucor* (one species), and *Akanthomyces* (one species). Thus, when a total of 381 fungal isolates were assigned to the eight genera, *Neurospora* was the most prevalent genus (135 CFU and 35.43%) among the eight genera, which was followed by *Chaetomium* (84 CFU and 22.05%), *Aspergillus* (46 CFU and 12.07%), *Penicillium* (44 CFU and 11.55%), *Mucor* (37 CFU and 9.71%), *Fusarium* (17 CFU and 4.46%), *Akanthomyces* (10 CFU and 2.62%), and *Alternaria* (8 CFU and 0.78%). When the 16 fungal isolates were further categorized, they belonged to five orders (*Neurospora* and *Chaetomium* in Sordariales, *Penicillium* and *Aspergillus* in Eurotiales, *Fusarium* and *Akanthomyces* in Hypocreales, *Alternaria* in Pleosporales, and *Mucor* in Mucorales).

**Figure 2:**
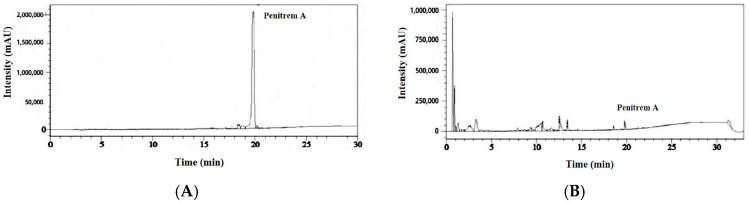
HPLC chromatograms of penitrem A. Chromatograms of (**A**) penitrem A standard solution (100 μg/mL) and (**B**) culture extracts of isolate P2. The retention time of the penitrem A peak was 19.7 min.

Then, we initially selected six isolates, which belong to *Penicillium* spp. and *Aspergillus* spp. and may produce patulin, and screened them for patulin production by HPLC analysis. However, none of the six fungal isolates produced patulin (Table S1). Thus, we screened three *Penicillium* spp. for production of other *Penicillium* associated mycotoxins such as penitrem A by HPLC analysis because it is one of the emerging mycotoxins found in food products. Of the three *Penicillium* spp., the *P. crustosum* isolate (P2) was able to produce a moderate level of penitrem A (0.62 ± 0.01 μg/mL) (Figure 2). We then designated the *P. crustosum* isolate (P2) as *P. crustosum* OM1. The morphology of *P. crustosum* OM1 on five types of media (PPAM, PDA, mYES4, mMEA, and mCYA) is shown in Figure S2.

We performed LC/MS/MS analysis to confirm the identity of penitrem A produced by *P. crustosum* OM1. The penitrem A standard was eluted at 15.982 min, and penitrem A in the fungal culture extracts was eluted at 15.933 min (Figure S3A,C). The mass-to-charge (*m*/*z*) ratio of the most abundant product ion [M+H]^+^ associated with the peak in the penitrem A standard was 634.2943 and the *m*/*z* ratio of the major product ion associated with the peak in the fungal culture extracts was 634.2934, and the MS profiles were well matched with each other (Figure S3B,D). These results confirmed the identity of penitrem A produced by *P. crustosum* OM1, which was detected by HPLC-UVD as described above.

### 3.2. Effects of Different Sucrose Contents on Penitrem A Production by P. crustosum OM1

Most fungi utilize sugar as a primary carbon source for their growth and mycotoxin production. The levels of sugar are also important factors for the mycotoxin production. Thus, we cultured *P. crustosum* OM1 on mYES agar plates containing two different sucrose concentrations (4 or 15%) at 22 °C to compare levels of penitrem A production by the fungal strain. The growth rate on mYES15 was higher than that on mYES4 as expected (Figure S4A). The fungal growth on mYES15 increased consistently until 16 days of incubation, whereas that on mYES4 increased slowly and reached a maximum after 16 days. Mycelial dry weight on mYES15 (1177.97 ± 22.27 mg) was approximately 2.5-fold higher than that on mYES4 (472.77 ± 22.01 mg) after 16 days of incubation. In contrast, the fungi produced a higher amount of penitrem A on mYES4 than mYES15 (Figure S4B). After 8 days of incubation, there were significant increases in penitrem A production on both media. The penitrem A production on mYES4 (3.41 ± 0.17 μg/mg dry weight) was approximately 2-fold higher than that on mYES15 (1.74 ± 0.01 μg/mg dry weight) after 16 days. These results indicate that the lower sucrose content (4%) was more favorable than the high sucrose content (15%) for the production of penitrem A by *P. crustosum* OM1 on mYES. Thus, we chose mYES4 instead of mYES15 for further experiments.

### 3.3. Influence of Temperature and Culture Media on the Growth of P. crustosum OM1 and Its Penitrem A Production

**Figure 3:**
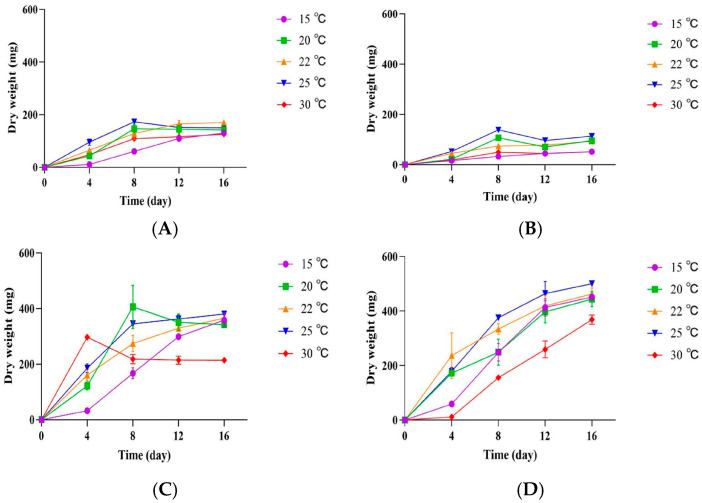
Growth of *P. crustosum* OM1 on 4 different agar plates (PDA, mYES4, mMEA, and mCYA) at 5 different temperatures (15, 20, 22, 25, and 30 °C). (**A**) Mycelial dry weight on PDA, (**B**) mycelial dry weight on mMEA, (**C**) mycelial dry weight on mCYA, and (**D**) mycelial dry weight on mYES4. The dry weight of mycelia was measured in triplicate. Data are expressed as the mean ± standard deviation.

To investigate the effects of temperature and culture media on the growth of *P. crustosum* OM1, we cultured the fungal strain on four different agar plates (PDA, mYES4, mMEA, and mCYA) under five different temperature conditions (15, 20, 22, 25, and 30 °C). The fungal growth showed similar patterns between PDA and mMEA (Figure 3A,B), and between mCYA and mYES4 (Figure 3C,D). The growth on both PDA and mMEA approached a plateau under all five temperature conditions after 8 days of incubation (Figure 3A,B). The growth rate on PDA was slightly higher (4%) than that on mMEA (173.27 ± 14.25 mg dry weight on PDA, 138.77 ± 15.62 mg dry weight on mMEA on the 8th day at 25 °C). However, the growth rates on mCYA and mYES4 were higher than those on PDA and mMEA. The growth on both mCYA and mYES4 was retarded at 30 °C, but was highest after 16 days of incubation under all temperature conditions except those on mCYA at 20 and 30 °C (Figure 3C,D). Although the growth on mCYA was highest at 20 °C after 8 days (406.23 ± 77.37 mg dry weight), it did not show a statistically significant difference relative to that at 25 °C after 16 days (380.80 ± 12.03 mg dry weight). Overall, of four different media, the fungal strain exhibited the highest growth rate on mYES4 after 16 days of incubation at 25 °C (500.97 ± 1.96 mg dry weight). These results imply that the optimum temperature for the growth of *P. crustosum* OM1 on four different types of media is 25 °C.

**Figure 4:**
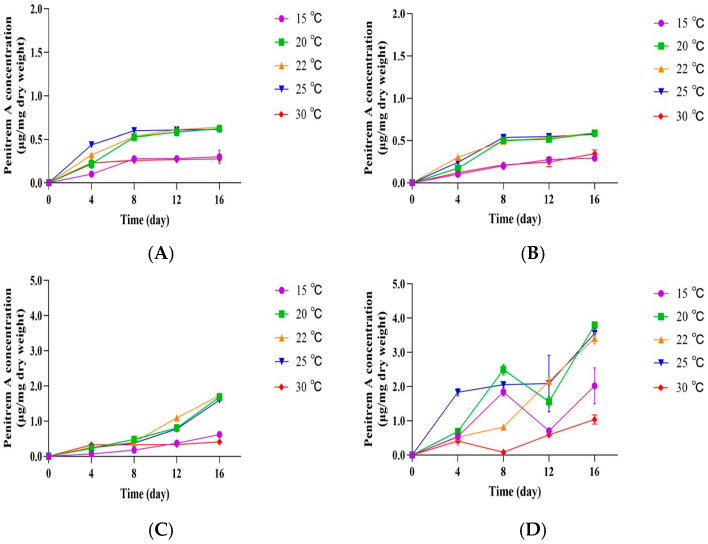
Penitrem A production by *P. crustosum* OM1 on 4 different agar plates (PDA, mYES4, mMEA, and mCYA) at 5 different temperatures (15, 20, 22, 25, and 30 °C). (**A**) Penitrem A level on PDA, (**B**) penitrem A level on mMEA, (**C**) penitrem A level on mCYA, and (**D**) penitrem A level on mYES4. The levels of penitrem A were measured in triplicate. Data are expressed as the mean ± standard deviation.

Next, we investigated the levels of penitrem A produced by *P. crustosum* OM1 on four different agar plates (PDA, mYES4, mMEA, and mCYA) under five different temperature conditions (15, 20, 22, 25, and 30 °C). The levels of penitrem A on both PDA and mMEA reached a plateau at under five temperature conditions after 8 days of incubation, which were similar to its growth patterns on the same media described above (Figure 4A,B). The penitrem A production on PDA was slightly better (12%) than that on mMEA (0.64 ± 0.01 μg/mg dry weight on PDA, 0.59 ± 0.01 μg/mg dry weight on mMEA on 16th day at 22 °C). Again, similarly to the growth patterns, the fungal strain produced higher levels of penitrem A on mCYA or mYES4 than on PDA or mMEA. In addition, the penitrem A production on both mCYA and mYES4 was highest after 16 days of incubation under all five temperature conditions (Figure 4C,D). The production on mYES4 at 20 °C (3.79 ± 0.09 μg/mg dry weight) was approximately 2.2-fold higher than that on mCYA at 22 °C (1.73 ± 0.03 μg/mg dry weight) after 16 days of incubation. Overall, the fungal strain showed high levels of penitrem A production under the temperature conditions between 20 and 25 °C after 16 days of incubation on all four different types of media. It produced the highest amounts of penitrem A on three different media (PDA, mMEA, and mCYA) at 22 °C, while it produced the highest amounts of penitrem A on mYES4 at 20 °C after 16 days. However, there was no statistically significant difference between the levels of penitrem A at 20 and 22 °C (3.79 ± 0.09 μg/mg dry weight and 3.41 ± 0.27 μg/mg dry weight, respectively). Thus, we chose 22 °C as an incubation temperature for further experiments. Also, we selected mYES4 medium as a culture medium, on which the fungal strain produced the highest amount of penitrem A.

### 3.4. Influence of pH on the Growth of P. crustosum OM1 and Its Penitrem A Production

**Figure 5:**
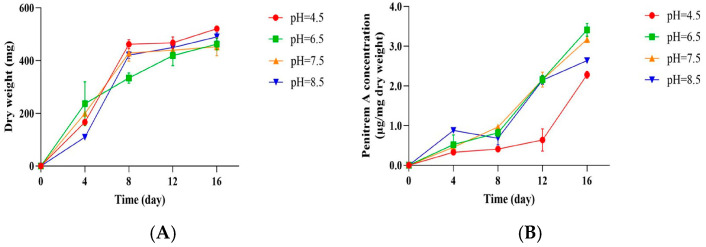
Growth of *P. crustosum* OM1 and its penitrem A production on mYES4 agar plates at 4 different pH (pH 4.5, 6.5, 7.5, and 8.5) and 22 °C. (**A**) Mycelial dry weight and (**B**) level of penitrem A. The levels of penitrem A and dry weight of mycelia were measured in triplicate. Data are expressed as the mean ± standard deviation.

It is known that environmental pH affects fungal growth and mycotoxin production due to the change in gene expression by pH regulatory systems and inhibition of intracellular enzyme activity [47,48,49]. Thus, to investigate the effect of pH on the growth of *P. crustosum* OM1 and its penitrem A production, we cultured the fungal strain on mYES4 agar plates under four different pH conditions (pH 4.5, 6.5, 7.5, and 8.5) at 22 °C. The fungal growth exhibited a typical increasing tendency under all four pH conditions (Figure 5A). The growth was highest at pH 4.5 (521.27 ± 5.39 mg/dry weight), followed by pH 8.5, 6.5, and 7.5 after 16 days of incubation. In contrast, the levels of penitrem A were higher at neutral pH (pH 6.5 and 7.5) than acidic or alkaline pH (pH 4.5 and 8.5) (Figure 5B). The fungal strain produced the highest level of penitrem A at pH 6.5 (3.41 ± 0.17 μg/mg dry weight), while it produced the lowest level of penitrem A at pH 4.5 (2.28 ± 0.09 μg/mg dry weight) after 16 days. These data imply that the optimum pH for the growth of *P. crustosum* OM1 on mYES4 at 22 °C is pH 4.5, whereas that for its penitrem A production on the same media at 22 °C is pH 6.5.

### 3.5. Growth of P. crustosum OM1 and Its Penitrem A Production on PPAM

Since *P. crustosum* OM1 was isolated from pears, it was necessary to assess its penitrem A production ability on PPAM, which is a simulated natural environment using pears. Thus, we evaluated the growth of *P. crustosum* OM1 and the amounts of penitrem A produced by the fungal strain on PPAM agar plates (pH 6.5) at 22° C. Fungal growth based on colony diameter increased steadily until the 16th day of incubation (81.00 ± 1.41 mm colony diameter), while that based on mycelial dry weight was highest on the 8th day (193.23 ± 12.85 mg dry weight), and thereafter it decreased slowly (Figure S5A). On the other hand, the fungal strain produced the highest amount of penitrem A on PPAM after 16 days of incubation (0.15 ± 0.01 μg mg/dry weight), but the level on the 12th day did not increase compared to that on the 8th day (Figure S5B), which was slightly different from that on mYES4 as shown in Figure 5A. As expected, the pH of PPAM gradually decreased over 16 days, but it seemed that the pH on the 16th day (3.76 ± 0.02) did not reach the level (below pH 3, see below) required for degradation of penitrem A (Figure S5B).

### 3.6. Influence of RH on the Growth of P. crustosum OM1 and Its Penitrem A Production on PPAM and mYES4

**Figure 6:**
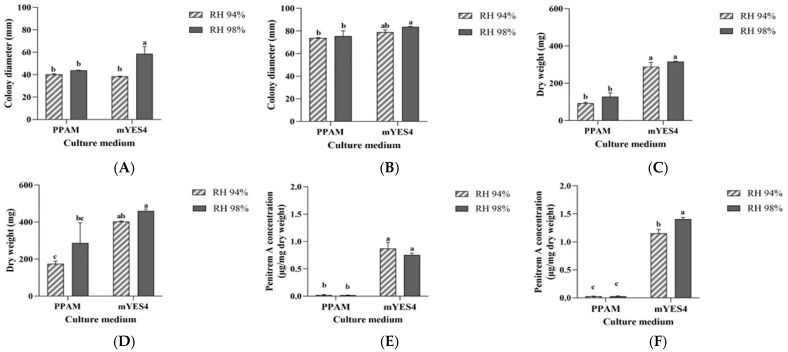
Growth of *P. crustosum* OM1 and its penitrem A production on PPAM (pH 6.5) or mYES4 (pH 6.5) agar plates under 2 different RH (94 and 98%) conditions at 22 °C. (**A**,**B**) Colony diameter after 5- and 10-day incubation, (**C**,**D**) mycelial dry weight after 5- and 10-day incubation, and (**E**,**F**) levels of penitrem A after 5- and 10-day incubation. The levels of penitrem A dry weight of mycelia, and colony diameter were measured in triplicate. Data are expressed as the mean ± standard deviation. Different letters indicate statistically significant differences (*p* < 0.05).

RH is another environmental parameter that affects fungal growth and mycotoxin production. Thus, we cultured *P. crustosum* OM1 on PPAM (pH 6.5) or mYES4 (pH 6.5) agar plates under two different RH (94 and 98%) conditions at 22 °C for 10 days. The pattern of the fungal colony diameters was similar to that of the mycelial dry weight on the same media under both RH conditions over 10 days (Figure 6A–D). Although the fungus showed similar colony diameters on PPAM and YES after 10 days of incubation, the mycelial dry weight on mYES4 (403.10 ± 2.67 mg under RH 94%, 460.07 ± 10.20 mg under RH 98%) was approximately 2-fold higher than that on PPAM (174.27 ± 14.27 mg under RH 94%, 286.83 ± 19.05 mg under RH 98%) (Figure 6B,D) as shown in Figure 5A and Figure S5A. We observed that the fungal strain grew more sparsely on PPAM than on mYES4, and this may be the reason for the difference (Figure S2A,C). However, the fungal strain produced similar amounts of dry weight on the same media under RH 94 and 98% after 10 days of incubation (Figure 6D), which is in agreement with the data based on colony diameters (Figure 6B). In contrast, the fungi produced the higher amount of penitrem A on mYES4 under RH 98% (1.41 ± 0.03 μg/mg dry weight) than that on the same media under RH 94% (1.15 ± 0.07 μg/mg dry weight) after 10 days of incubation (Figure 6F). However, the fungal strain produced very low levels of penitrem A on PPAM under both RH 94 and 98% (0.03 ± 0.02 μg/mg dry weight and 0.03 ± 0.01 μg/mg dry weight, respectively) compared to those on mYES4 under the same RH. These levels of penitrem A are consistent with the above results shown in Figure 5B and Figure S5B. Thus, as expected, these data imply that the higher percentage of RH (98%) was more favorable than the lower RH (94%) condition for the production of penitrem A by *P. crustosum* OM1.

Considering the above results, our data demonstrated that the three key environmental factors such as temperature, pH, and RH significantly affected the growth of *P. crustosum* OM1 and its penitrem A production.

### 3.7. Growth of P. crustosum OM1 and Its Penitrem A Production in Liquid Media

**Figure 7:**
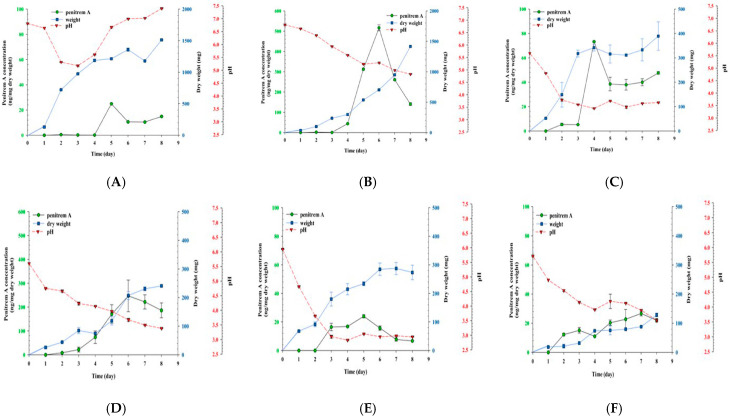
Growth of *P. crustosum* OM1 and its penitrem A production in 3 different liquid media (mYES4, PDB, and mMEB) at 22 °C with or without shaking. (**A**) mYES4 with shaking, (**B**) mYES4 without shaking, (**C**) PDB with shaking, (**D**) PDB without shaking, (**E**) mMEB with shaking, and (**F**) mMEB without shaking. The levels of penitrem A and dry weight of mycelia, and pH were measured in triplicate. Data are expressed as the mean ± standard deviation.

As described above, our data showed that *P. crustosum* OM1 produced the highest, middle, and lowest levels of penitrem A on mYES4, PDA, and mMEA agar plates, respectively. Thus, to compare levels of penitrem A produced by *P. crustosum* OM1 in liquid media, the fungal strain was cultured in three different types of liquid media (mYES4, PDB, and mMEB) under static or agitation conditions because mycotoxins such as patulin are produced at higher levels by *Penicillium* spp. under static conditions than agitation conditions [50]. Since previous studies reported that 97–99% of total penitrem A was detected in mycelia of *P. crustosum* instead of culture broth [22,40,41], we initially compared levels of penitrem A in mycelia of *P. crustosum* OM1 with those in its culture filtrate after 7 days of incubation under static conditions at 22 °C. As shown in Table S2, more than 95% of total penitrem A was detected in the mycelia when it was cultured in all three types of media. Thus, we extracted penitrem A from mycelia to analyze the mycotoxin. After the penitrem A was not detected until 3–4 days, the level increased in mYES4 under both conditions (Figure 7A,B). It reached the highest level (24.95 ± 0.01 ng/mg dry weight) in mYES4 under agitation conditions on the 5th day, while it reached the highest level (516.84 ± 14.50 ng/mg dry weight) in mYES4 under static condition on the 6th day. It indicates that the level of penitrem A in mYES4 under static conditions was 20-fold higher than that in mYES4 under agitation conditions. The mycelial dry weight showed a similar pattern to each other under both culture conditions although the dry weight in mYES4 with agitation increased more rapidly than that in mYES4 without agitation (Figure 7A,B). Interestingly, after the pH in mYES4 with shaking dropped from 6.8 to 5.3 until 3 days of incubation, it increased consistently to pH 7.5 on the 8th day. This result is similar to that from one previous study, in which pH was dropped from 7 to 4.5 until 3 days, which was followed by an increase to near neutral pH on the 6th day when a *P. crustosum* strain was cultured in CYB with shaking at 22 °C [25]. Also, *P. crustosum* OM1 produced the highest amount of penitrem A (73.30 ± 0.46 ng/mg dry weight) in PDB with agitation on 4th day, while it produced the highest amount of penitrem A (247.04 ± 66.37 ng/mg dry weight) in PDB without agitation on the 6th day (Figure 7C,D). The level of penitrem A production was 3.4-fold higher in PDB under static conditions than in PDB under agitation conditions. The mycelial dry weight was highest on the 8th day under both culture conditions, but the dry weight in PDB with agitation (389.2 ± 8.55 mg) exhibited a 1.6-fold higher level than that in PDB without agitation (240.95 ± 3.89 mg) on the same day (Figure 7C,D). In addition, the highest amount of penitrem A produced by the fungal strain in mMEB during 8 days of culture was similar to each other under both culture conditions (23.98 ± 1.09 ng/mg dry weight in mMEB with shaking on the 5th day, 26.72 ± 1.66 ng/mg dry weight in mMEB without shaking on the 7th day) (Figure 7E,F). The mycelial dry weight also exhibited a similar pattern to each other under both culture conditions, but the dry weight in mMEB with agitation (287.35 ± 21.28 mg on the 7th day) was 2-fold higher than that in mMEB without agitation (128.45 ± 6.58 mg on the 8th day) (Figure 7E,F). The pH in PDB and mMEB decreased until 3–3.5 and it was maintained under both culture conditions (Figure 7C–F). Thus, the fungus produced the highest level of penitrem A in mYES4 under static conditions, whereas it produced the lowest level of penitrem A in mMEB under agitation conditions. Overall, our results demonstrated that level of penitrem A produced by *P. crustosum* OM1 was highest in mYES4 under static conditions, whereas the level was lowest in mMEB under agitation conditions among the three types of media (mYES4, PDB, and mMEB) (Figure 7). Also, the data exhibited that its dry weight was highest in mYES4 with agitation among the three types of media.

### 3.8. Relative Gene Expression Analysis by RT-qPCR

**Figure 8:**
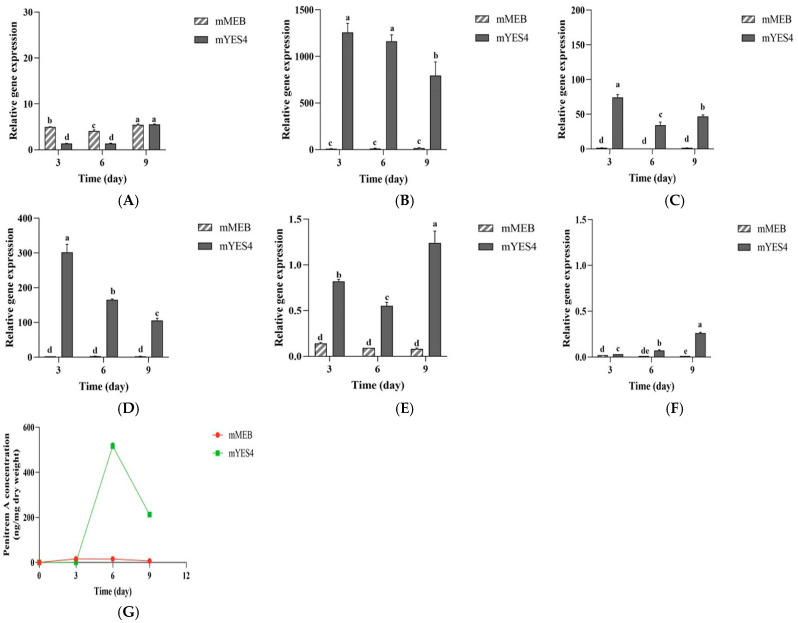
Time course of relative expression of putative penitrem A biosynthetic genes in *P. crustosum* OM1 and levels of its penitrem A production in mYES4 (penitrem A conducive) or mMEB (penitrem A non-conducive) medium. Relative expression levels of (**A**) *ptmB*, (**B**) *ptmJ*, (**C**) *ptmK*, (**D**) *ptmO*, (**E**) *ptmS*, (**F**) *ptmT*, and (**G**) penitrem A levels in mYES4 or mMEB. The expression levels of 6 genes and levels of penitrem A were measured in triplicate. The expression levels were normalized to that of β-tubulin gene. Data are expressed as the mean ± standard deviation. Different letters indicate statistically significant differences (*p* < 0.05).

The relative expression levels of six penitrem A biosynthetic genes (*ptmB*, *ptmJ*, *ptmK*, *ptmO*, *ptmS*, and *ptmT*) in the penitrem A gene cluster were analyzed by RT-qPCR when *P. crustosum* OM1 was cultured in mYES4 (penitrem A conducive medium) or mMEB (penitrem A non-conducive medium). Of the six genes, the relative expression levels of four penitrem A biosynthetic genes (*ptmJ*, *ptmK*, *ptmO*, and *ptmS*) increased in mYES4 on the 3rd day, and thereafter the levels gradually decreased until 9 days of incubation or remained relatively constant after the 6th day (Figure 8B–E). However, the relative expression levels of the four genes remained low in mMEB over 9 days. In contrast, although the relative expression levels of *ptmB* increased slightly in mYES4 after 9 days of incubation, they remained relatively constant in both mYES4 and mMEB during cultures (Figure 8A). Also, *ptmT* exhibited similar expression patterns to *ptmB* in both mYES4 and mMEB although the relative expression level of *ptmT* was lower in mMEB than in mYES4 on the 9th day (Figure 8A,F).

HPLC analyses showed that *P. crustosum* OM1 produced low levels of penitrem A in mMEB (15.69 ± 1.52 ng/mg dry weight on the 6th day), while it produced significantly increased levels of penitrem A in mYES4 (516.84 ± 14.50 ng/mg dry weight on the 6th day) although the level of penitrem A in mYES4 decreased to approximately 40% on the 9th day (212.66 ± 8.79 ng/mg dry weight) (Figure 8G), which is in agreement with the data shown in Figure 7. These results are also consistent with those of RT-qPCR analysis described above, indicating that the four penitrem A biosynthetic genes were up-regulated in mYES4 (penitrem A conducive medium), whereas they were not up-regulated in mMEB (penitrem A non-conducive medium).

## 4. Discussion

A number of studies documented that environmental conditions such as temperature, pH, RH, and water activity (a_w_; the degree of water available for microbes to use) influence fungal growth and mycotoxin production in food [51,52]. We initially attempted to isolate patulin-producing fungal strains from pears, but were not able to isolate patulin producers. Hence, we focused on another mycotoxin penitrem A-producing fungal strain.

Penitrem A, an emerging neurotoxin, is found in food such as cereal grains, meat and dairy products, and fruits [3,24]. Penitrem A intoxication has been associated with ingestion of cereal grains, nuts, and dairy products in humans and animals [7,8,10,11]. Few studies have reported on environmental conditions for penitrem A production by *P. crustosum*. Thus, in the current study, we isolated *P. crustosum* OM1 from pears, tested it for penitrem A production, and investigated the impacts of the three major physicochemical parameters (temperature, pH, and RH) on the growth of *P. crustosum* OM1 and its penitrem A production.

Our results showed that 16 fungal strains, which were isolated from pears, belong to *Neurospora*, *Penicillium*, *Aspergillus*, *Fusarium*, *Chaetomium*, *Alternaria*, *Mucor*, and *Akanthomyces* genera. This taxonomic classification is in line with the results on fungal strains isolated from pears in other studies [53,54,55]. A previous study described that the most common causative pathogens isolated from Asian pears (cultivars, Zaosu and Yali) in China were *Penicillium* spp., *Alternaria* spp., *Botrytis* spp., and *Mucor* spp. [53]. Another previous study from Malta documented that common fungal pathogens found on several different pear cultivars were *Penicillium* spp., *Aspergillus* spp., *Botrytis* spp., *Mucor* spp., *Alternaria* spp., *Rhizopus* spp., *Phialophora* spp., *Cladosporium* spp., and *Neofabrea* spp. [54]. However, these results showed a slight difference from our data. The discrepancy may have resulted from different pear varieties, geographical location, or climate.

In this study, when we analyzed the identity of penitrem A produced by *P. crustosum* OM1 using LC/MS/MS, we found another substance in the fungal culture extracts, which was eluted at 14.634 min (Figure S3E). The *m*/*z* ratio of the most abundant product ion [M+H]^+^ associated with the substance peak was 390.1928, and its molecular formula was C_22_H_23_N_5_O_2_ (Figure S3F). We assume that the substance is roquefortine C because it matched well with roquefortine C (98% score) in METLIN PCDL database, which was connected to Agilent LC/Q-TOF, and it was reported to be produced by *P. crustosum* in previous studies [24,25,26,40,56]. Sonjak and co-workers described that all 121 *P. crustosum* strains, which were isolated from Arctic and non-Arctic regions, produced roquefortine C along with penitrem A, and concluded that *P. crustosum* produces both metabolites concurrently [24], which is in agreement with our results.

Our data showed that *P. crustosum* OM1 produced 2-fold higher amounts of penitrem A on mYES containing low sucrose (4%) than that containing high sucrose (15%). It is in line with other previous studies [22,41]. El-Banna and colleagues reported that the level of penitrem A in a *P. crustosum* strain decreased by approximately 50% in SPS medium containing 16% sucrose relative to the same medium containing 2% sucrose under static conditions [22]. Another study documented that one *P. crustosum* strain (formerly misidentified as *P. commune* but re-identified by Frisvad [57]) produced approximately 5-fold higher levels of penitrem A in YES4 than in YES15 when it was cultured under static conditions at room temperature [41].

Our results also exhibited high levels of penitrem A production under the temperature conditions between 20 and 25 °C on all four different types of media (mYES4, PDA, mMEA, and mCYA). It is consistent with the results from other previous studies [5,22,26,41]. Kalinina and co-workers reported that penitrem A production by *P. crustosum* CBS 483.75 was highest at 22 °C on CYA after 14 days of incubation, followed by 24 and 20 °C [5]. El-Banna and collaborators showed the optimum temperature for penitrem A production at 25 °C after 3-week culture of one *P. crustosum* strain on SPS medium under static conditions [22]. Another study obtained similar results to those from El-Banna and collaborators, and the authors described that the optimum temperature for penitrem A production was 25 °C when they cultured a *P. crustosum* strain on rice under three temperature conditions (10, 15, and 25 °C) [26]. Also, Wagerner and colleagues documented that the optimum temperature for penitrem A production on YES4 by one *P. crustosum* strain (misidentified as *P. commune*) was 20 °C under static conditions after 4 weeks incubation [41].

In addition, our data showed that *P. crustosum* OM1 produced higher amounts of penitrem A on mCYA or mYES4 than on PDA or mMEA and that it produced much higher levels on mYES4 than on mCYA at 22 °C. The former is similar to those from previous studies [5,22,41]. Kalinina and co-workers described that *P. crustosum* CBS 483.75 produced higher levels of penitrem A on CYA and YES than on MEA and PDA at 25 °C after 14 days although we used slightly modified composition in three media (mCYA, mMEA, and mYES) [5]. However, in their study, higher amounts of penitrem A were produced by the strain on CYA than on YES, which is different from higher levels of penitrem A production by *P. crustosum* OM1 on mYES than on mCYA in our study. This discrepancy may have resulted from the use of a different *P. crustosum* strain or the slightly different media composition in their study (YES containing 3% glucose). Also, in El-Banna and Leistner’s study, higher levels of penitrem A were produced by a *P. crustosum* strain on CYB than on MEB at 25 °C under static conditions although they used slightly different compositions in both media (CYB containing 2% yeast extract, MEB containing only malt extract) from those in our media (mCYA, mMEA) [22]. In another study, one *P. crustosum* strain (misidentified as *P. commune*) produced a 1.4-fold higher level of penitrem A in YES4 than in CYB containing 2% yeast extract under static conditions at room temperature [41]. It has been documented that YES media containing sucrose are generally favorable for production of secondary metabolites including mycotoxins by *Penicillium* spp. relative to the media containing the other carbon sources [58]. Kumar and colleagues reported that *Penicillium expansum* produced a higher level of patulin, a mycotoxin, on solid media containing sucrose than glucose or fructose and that as sucrose concentrations were higher, the levels of patulin production were reduced due to decreased expression levels of LaeA, a global transcription factor of secondary metabolism [59]. Thus, these might be the reasons why our study showed higher penitrem A production by *P. crustosum* OM1 on mYES4 than on mYES15 and on mYES4 than on mMEA (2% glucose) and PDA (2% glucose). Also, in our study, higher penitrem A production on mYES4 (2% yeast extract) than on mCYA (3% sucrose, 0.5% yeast extract) might have been due to the effect of yeast extract because it is rich in a variety of trace elements such as vitamins and minerals that can be used as cofactors of many enzymes [60] and it was reported that an addition of yeast extract to synthetic media is crucial for penitrem A production by *P. crustosum* [5]. In addition, when we analyzed fungal growth based on mycelial dry weight on PPAM (pH 6.5) and mYES4 (pH 6.5), the growth on PPAM was slightly different from that on mYES4. The dry weight on PPAM after 16 days of incubation (162.50 ± 4.46 mg dry weight) was approximately 3-fold lower than that on mYES4 after the same period of incubation (462.77 ± 22.01 mg/dry weight). We assume that the decreased dry weight on PPAM was due to insufficient nutrients on the media (insufficient growth factors from no peptone or yeast extract in pear puree [61]) compared to mYES4.

Several previous studies have shown that neutral pH conditions were more supportive of penitrem A production by *P. crustosum* than acidic or alkaline conditions [5,22], although the fungus had generally optimal growth between pH 4.5 and 9 [62]. El-Banna and Leistner described that a *P. crustosum* strain produced higher amounts of penitrem A on SPS medium at near neutral pH (pH 5.7–6.0) than acidic or alkaline pH [22]. Another study reported that the highest amount of penitrem A was produced by *P. crustosum* CBS 483.75 at pH 6 among pH 3, 6, and 9 on CYA after 21 days [5]. These data are in agreement with those from our study. In the current study we showed that the optimum pH for penitrem A production by *P. crustosum* OM1 on mYES4 at 22 °C was pH 6.5 although the fungal strain grew well in broad pH ranges between pH 4.5 and 8.5 on the same media at 22 °C, which is in line with the results from Pitt’s study [62]. Considering the above data, our results demonstrated that environmental temperature and pH have a significant influence on the growth of *P. crustosum* OM1 and its penitrem A production.

Another environmental parameter RH also affects fungal growth and mycotoxin production [41,63]. Rundberget and collaborators described that a_w_ is an important parameter that influences penitrem production and that penitrem A production decreases rapidly below a_w_ 0.99 [26]. In our study, *P. crustosum* OM1 grew well and produced high amounts of penitrem A on mYES4 under RH 94%, which were similar to those under RH 98%, while it produced very low levels of penitrem A on PPAM under both RH 94 and 98% although its growth was comparable to those on mYES4 as described above. Wagener and colleagues reported that one *P. crustosum* strain (misidentified as *P. commune*) produced much lower amounts of penitrem A on cottonseed under RH 95% than under RH 99% [41]. Another study documented that below a_w_ 0.958, they detected growth of *P. crustosum* CBS 483.75 but did not detect any penitrems, including penitrem A on CYA after 21 days [5], which slightly differed from those of our study. This may have been due to the use of different media. Our results also showed that the levels of penitrem A on PPAM did not increase on the 10th day compared to the 5th day and were very low at 22 °C under both RH 94 and 98% conditions. Thus, it is not likely that penitrem A production by *P. crustosum* OM1 on pears occurs during storage at lower temperature than 22 °C such as 4 °C. Overall, our data demonstrated that the three key physicochemical parameters such as temperature, pH, and RH strongly influenced the growth of *P. crustosum* OM1 and its penitrem A production.

On the other hand, Rundberget and co-workers documented that 10–15% of penitrem A was converted to thonitrem A (its structurally related analog) at pH 2–3 after 24 h incubation since penitrems are unstable at low pH [26]. In our study, the pH in PDB and mMEB decreased until 3–3.5 and it was maintained under both agitation and static conditions. In particular, the pH in mMEB under the agitation condition was maintained at near pH 3 after 3 days of incubation, and the level of penitrem A on the 8th day decreased by approximately 4-fold relative to that on the 5th day. The penitrem A in mMEB may have been converted to another metabolite such as thonitrem A during the culture at pH 3. These results suggest that acidic conditions are not favorable for penitrem A production by *P. crustosum* OM1. Also, Sumarah and colleagues showed that penitrem A production started with the maximum rate after carbon sources such as glucose or fructose, which was converted from sucrose, are depleted in CYB culture of one *P. crustosum* strain [25]. It is in line with our study, in which *P. crustosum* OM1 started to produce penitrem A after 2–3 days of incubation. As expected, our data showed that *P. crustosum* OM1 produced the highest level of penitrem A in mYES4 under static conditions, whereas it had the highest level of dry weight in mYES4 under agitation conditions among three types of media (mYES4, PDB, and mMEB). It seems that mYES4 containing sucrose supported penitrem A production by *P. crustosum* OM1 as described above and that the increased fungal cell mass but not penitrem A production was facilitated by increased oxygen supply under agitation conditions [64,65].

Lastly, RT-qPCR analysis showed that the relative expression levels of four penitrem A biosynthetic genes (*ptmJ*, *ptmK*, *ptmO*, and *ptmS*) in *P. crustosum* OM1 increased in mYES4 (penitrem A conducive) on the 3rd day, and thereafter the levels gradually decreased until 9 days of incubation or remained relatively constant after the 6th day. However, they were maintained very low in mMEB (penitrem A non-conducive) compared to those in mYES4. Moreover, HPLC analyses showed that the fungal strain produced remarkably increased levels of penitrem A in mYES4, whereas it produced small amounts of penitrem A in mMEB. These results indicate that the relative expression of the four penitrem A biosynthetic genes (*ptmJ*, *ptmK*, *ptmO*, and *ptmS*) were up-regulated in mYES4 medium, while they were not up-regulated in mMEB medium. It strengthens the fact that mYES4 containing sucrose supports more penitrem A production than mMEB containing glucose as described above. Also, the HPLC analysis results demonstrated that the level of penitrem A decreased in mYES4 after 6 days of incubation, which is consistent with the decreased expression levels of *ptmJ*, *ptmK*, and *ptmO*, and the results from one previous study [40]. Mantle and collaborators described that the level of penitrem A was reduced after 8 days of incubation when they cultured a *P. crustosum* strain in CYB under static conditions at 27 °C [40]. However, intriguingly, in this study as described above, the levels of penitrem A in mYES4 agar plates increased continuously even after 6 days. These results might indicate different patterns of gene expression between cultures in the liquid and solid media due to more rapid diffusion of H^+^ to lower pH in liquid media. In addition, the relative expression levels of *ptmB* or *ptmT* in mYES4 were similar to those in mMEB during 9 days of culture. Several previous studies established the penitrem A biosynthetic pathway [16,18]. Based on the penitrem A biosynthetic pathway, our results indicate that *ptmS*, which encodes NmrA-like family transcription factor, is constitutively expressed in mYES4 to induce the expression of the three penitrem A biosynthetic genes (*ptmJ*, *ptmK*, and *ptmO*) during penitrem A biosynthesis, whereas *ptmB* (encodes indole-diterpene cyclases) or *ptmT* (encodes major facilitator superfamily transporters) exhibited relatively constant expression in both mYES4 and mMEB.

## 5. Conclusions

In the current study, we evaluated the influence of three major physicochemical parameters (temperature, pH, and RH) on the growth of *P. crustosum* OM1 isolated from pears and its penitrem A production. Our results demonstrated that the environmental factors had a significant impact on the penitrem A production by the fungal strain and its growth. It showed that the highest growth rate of *P. crustosum* OM1 was produced at 25 °C and pH 4.5 on mYES4 under RH 98%, whereas the highest level of penitrem A was produced at 22 °C and pH 6.5 on the same media under RH 98%. In addition, the fungal strain produced very low levels of penitrem A on PPAM at 22 °C under both RH 94 and 98% compared to those on mYES4 under the same RH. These data suggest that penitrem A production on pears will be controlled by storage below 22 °C and RH 94%. In addition, the relative expression levels of *ptmJ*, *ptmK*, *ptmO*, and *ptmS* were up-regulated in mYES4 medium, whereas they were not up-regulated in mMEB medium. These results will also help understand the physiological characteristics of *P. crustosum* OM1 on media containing different carbon sources. In conclusion, our study will provide new insights into finding environmental conditions to eliminate the occurrence of penitrem A contamination by *P. crustosum* on fresh fruits such as pears during storage.

## Figures and Tables

**Table 1:** Primer sequences for RT-qPCR analysis of 6 penitrem A biosynthetic genes.

Gene	Orientation	Primer Sequence (5′ → 3′)
*ptmB*	Forward	GTCCTGCACTGTGGCTTTTG
	Reverse	CTCCACAAAACCAGAGGGCT
*ptmJ*	Forward	ACTCCAGTCTGCACCCATTG
	Reverse	TCACGCAGAGACTCAGGAGA
*ptmK*	Forward	CTGACCCAGCAGATCAGACG
	Reverse	CGTGGTGCCCAATAGACCAT
*ptmO*	Forward	CCGCAAAACACGAGAAGAGC
	Reverse	TGGTTCAATGTCGGCGTGTA
*ptmS*	Forward	CCCAAGGGCACTACTTTGGT
	Reverse	AAAGAGGACGATTGCTGCGA
*ptmT*	Forward	GGAATCATGTCGGGCATTGC
	Reverse	TGAAGACCACAGTGCCGATC
*β-tubulin*	Forward	GTCCAACGACAGGAAACCGA
	Reverse	CAGGCTCCAAATCGACGAGA

## Data Availability

All data generated or analyzed during this study are included in this published article and supplementary material.

## Supplementary Materials

The following supporting information can be downloaded at https://www.mdpi.com/article/10.3390/jof11100741/s1; Figure S1: Calibration curve of (A) patulin and (B) penitrem A standard solutions for quantification by HPLC; Figure S2: Morphology of *P. crustosum* OM1 on 5 different culture media; Figure S3: Extracted ion chromatograms (EIC) and mass (MS) spectra of penitrem A and roquefortine C; Figure S4: Growth of *P. crustosum* and the levels of penitrem A produced by the fungal strain on mYES agar plates containing 2 different sucrose concentrations (4 or 15%); Figure S5: Growth of *P. crustosum* OM1 and its penitrem A production on PPAM (pH 6.5) agar plates at 22 °C; Table S1: Levels of penitrem A or patulin produced by fungal isolates and their scientific names identified by BLAST-based analysis; Table S2: Percentages of penitrem A detected in 2 different parts of *P. crustosum* OM1 after culture in 3 different liquid media for 7 days.

## Abbreviations

The following abbreviations are used in this manuscript:

GABA	ϒ-aminobutyric acid
ITS	Internal transcribed spacer
PPAM	Pear puree agar medium
RH	Relative humidity
