# Supplementary material for: Effects of Temperature, pH, and Relative Humidity on Growth of Penicillium crustosum OM1 Isolated from Pears and Its Penitrem A Production
Source: J Fungi (Basel). 2025 Oct 16;11(10):741. doi: 10.3390/jof11100741 (PMC12564995; doi:10.3390/jof11100741)
Supplement: Supplementary file 1 [file jof-11-00741-s001.zip › jof-3894279-supplementary.pdf]

Supplementary Figures

A.

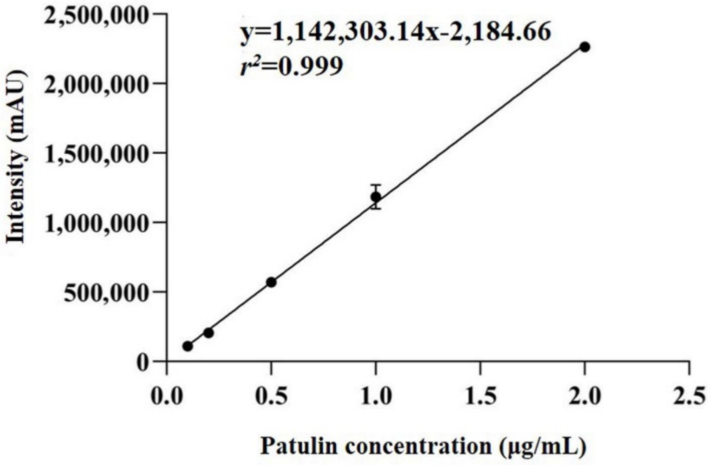

B.

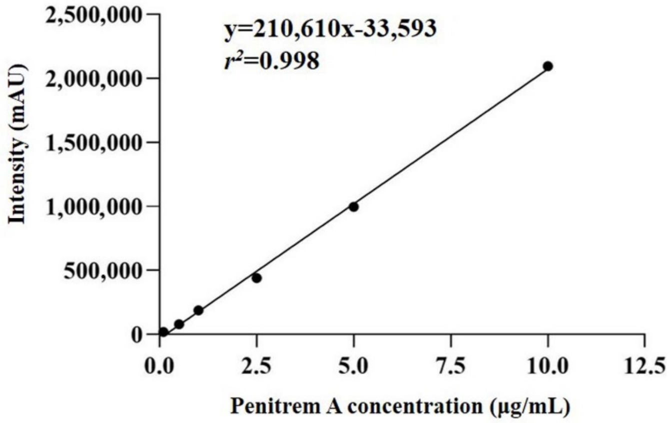

Figure S1. Calibration curve of (A) patulin and (B) penitrem A standard solutions for quantification by HPLC. Patulin standard solutions were prepared in the range of 0.1, 0.2, 0.5, 1.0, and 2.0 μg/mL, while penitrem A standard solutions were prepared in the range of 0.1, 0.5, 1.0, 2.5, 5.0, and 10.0 μg/mL. Each solution was analyzed by HPLC-UVD in triplicate.

A.

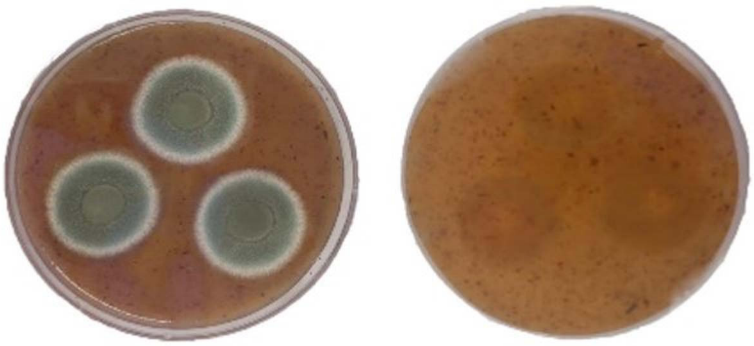

B.

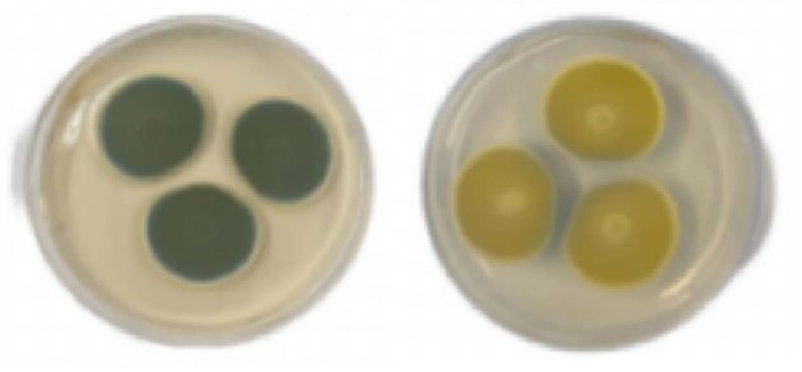

C.

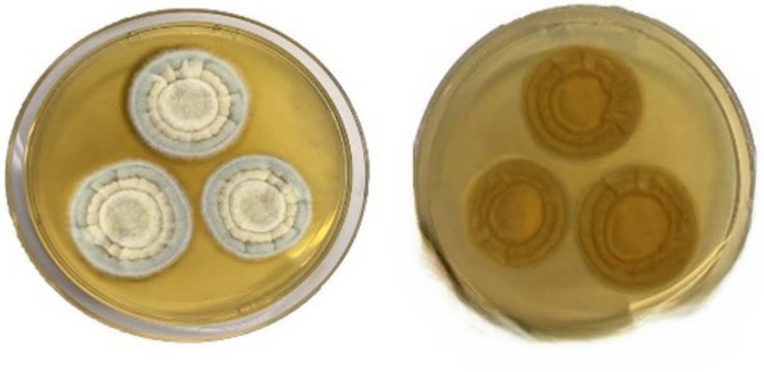

D.

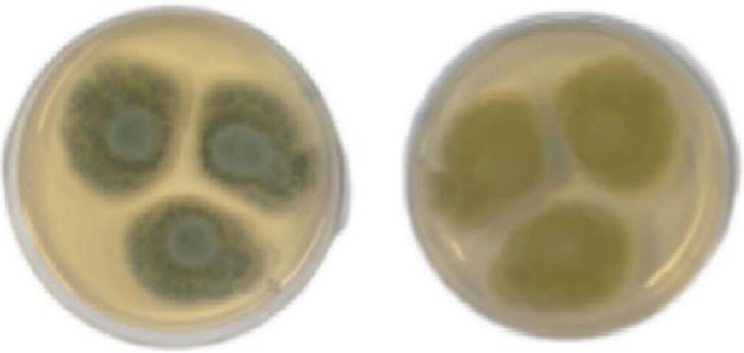

E.

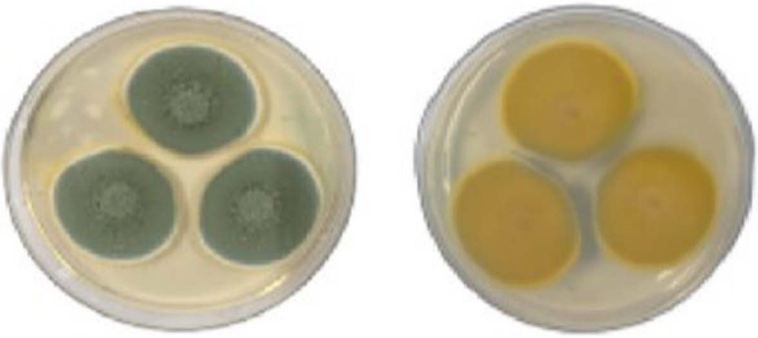

F.

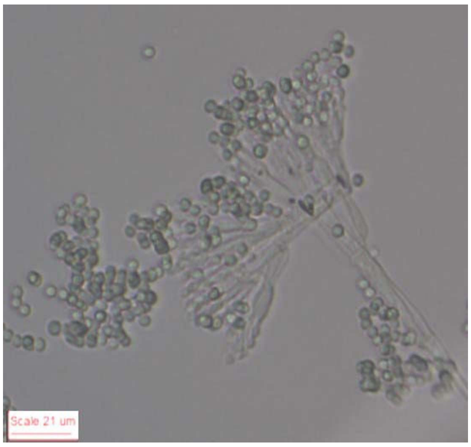

G.

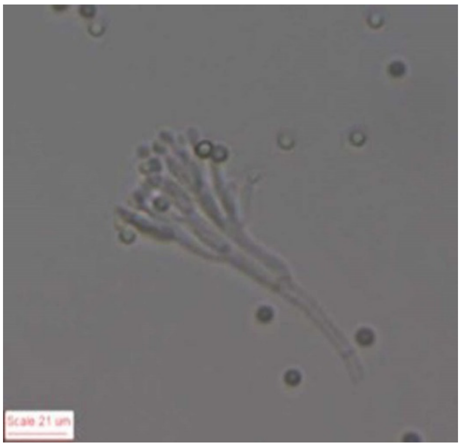

H.

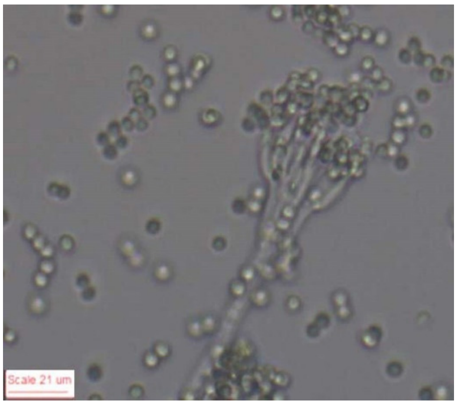

Figure S2. Morphology of *P. crustosum* OM1 on 5 different culture media. (A) Fungal colonies on PPAM agar plates, (B) fungal colonies on PDA agar plates, (C) fungal colonies on mYES4 agar plates, (D) fungal colonies on mMEA agar plates, (E) fungal colonies on mCYA agar plates, (F) conidiophore and conidiospores on PPAM agar plates (400x), (G) conidiophores and conidiospores on PDA agar plates (400x), and (H) conidiophores and conidiospores on mMEA agar plates (400x). Left photographs show top views, while right photographs show bottom views in (A), (B), (C), (D), and (E). Scale bar, 21 μm.

A.

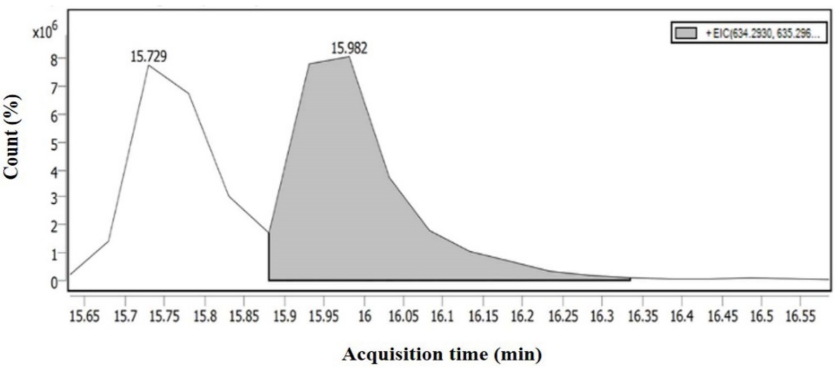

x10^6
15.729
15.982
+EIC(634.2930, 635.296...
Count (%)
Acquisition time (min)

B.

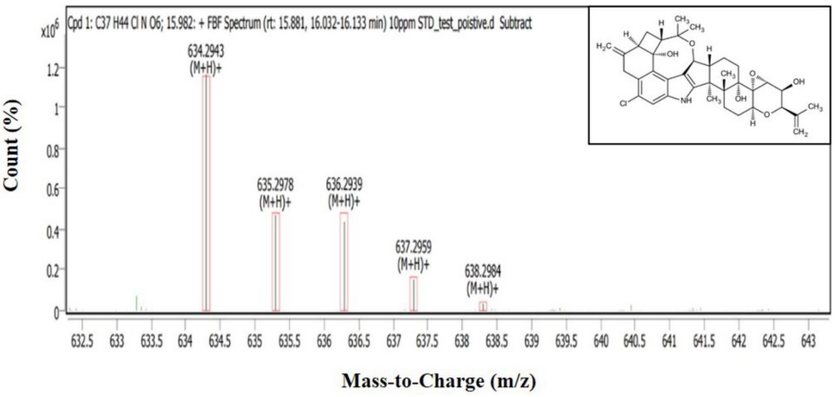

Cpd 1: C37 H44 Cl N O6; 15.982: + FBF Spectrum (rt: 15.881, 16.032-16.133 min) 10ppm STD_test_poistive.d Subtract
x10^6
634.2943 (M+H)+
635.2978 (M+H)+
636.2939 (M+H)+
637.2959 (M+H)+
638.2984 (M+H)+
Count (%)
Mass-to-Charge (m/z)

C.

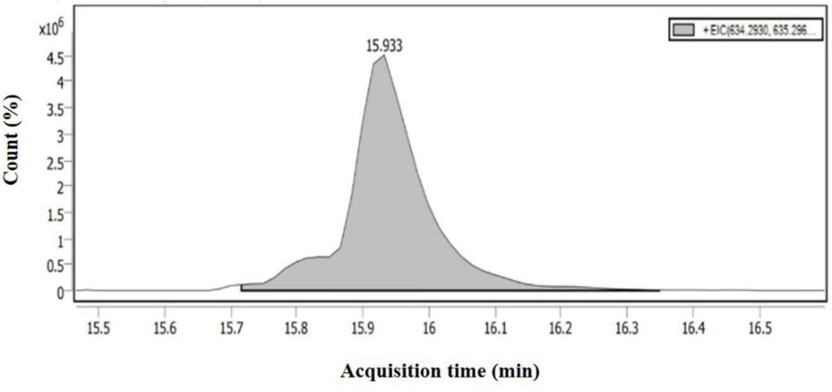

x10^6
15.933
+EIC(634.2930, 635.296...
Count (%)
Acquisition time (min)

D.

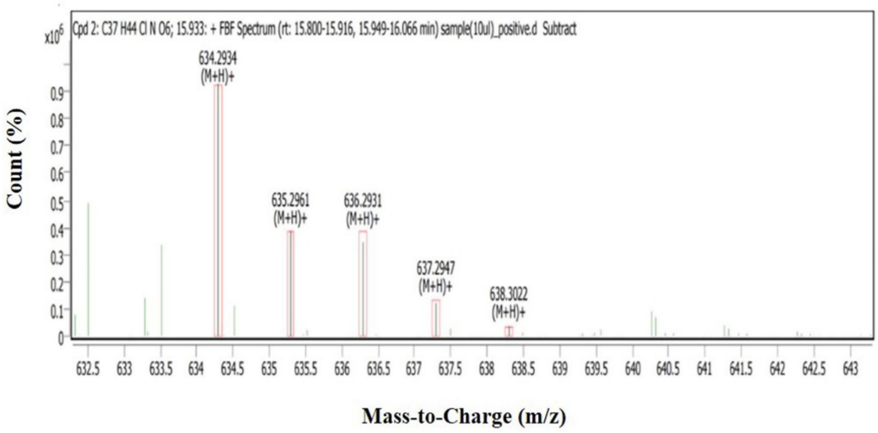

E.

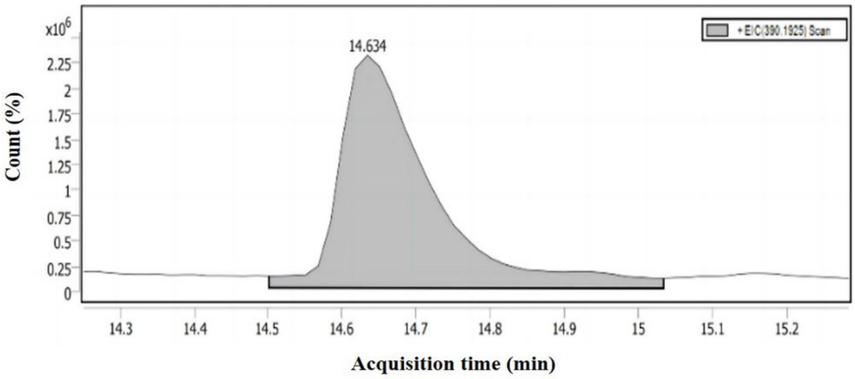

F.

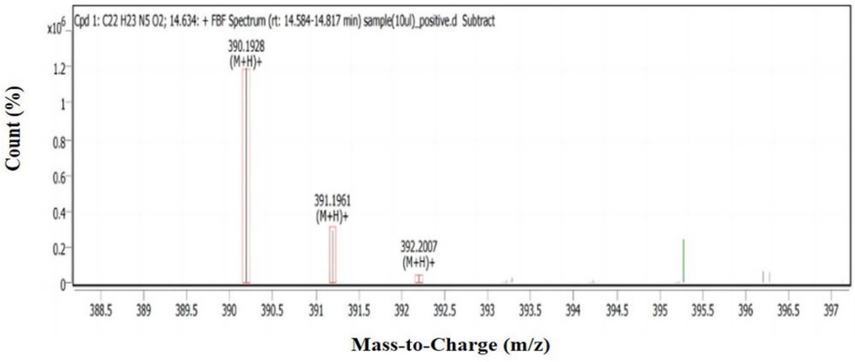

Figure S3. Extracted ion chromatograms (EIC) and mass (MS) spectra of penitrem A and roquefortine C. (A) EIC and (B) MS spectrum for penitrem A in a penitrem A standard solution (2 μg/mL), and (C) EIC and (D) MS spectrum for penitrem A and (E) EIC and (F) MS spectrum for roquefortine C in culture extracts of *P. crustosum* OM1. (Inset) penitrem A structure.

A.

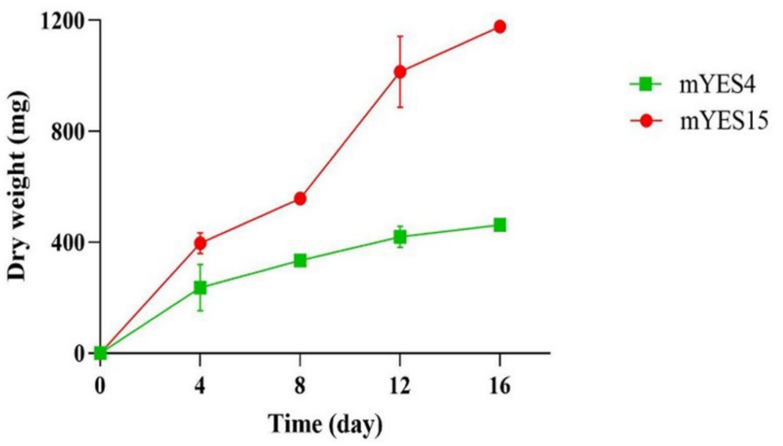

B.

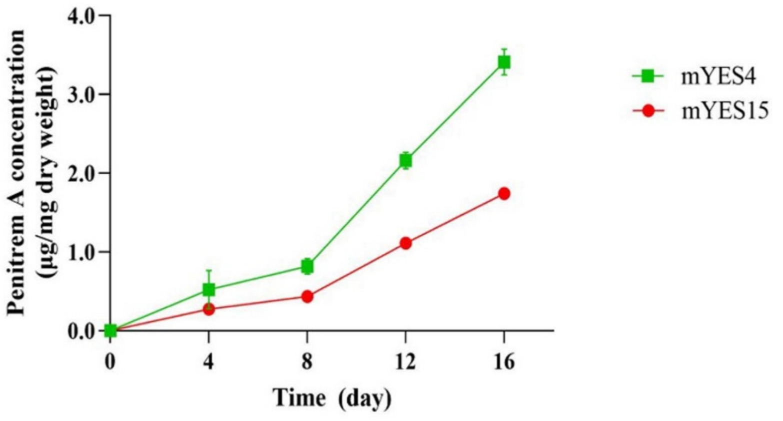

Figure S4. Growth of *P. crustosum* OM1 and the levels of penitrem A produced by the fungal strain on mYES agar plates containing 2 different sucrose concentrations (4 or 15%). (A) Dry weight of mycelia and (B) levels of penitrem A on mYES4 and mYES15. The dry weight of mycelia and levels of penitrem A were measured in triplicate. Data are expressed as the mean ± standard deviation.

A.

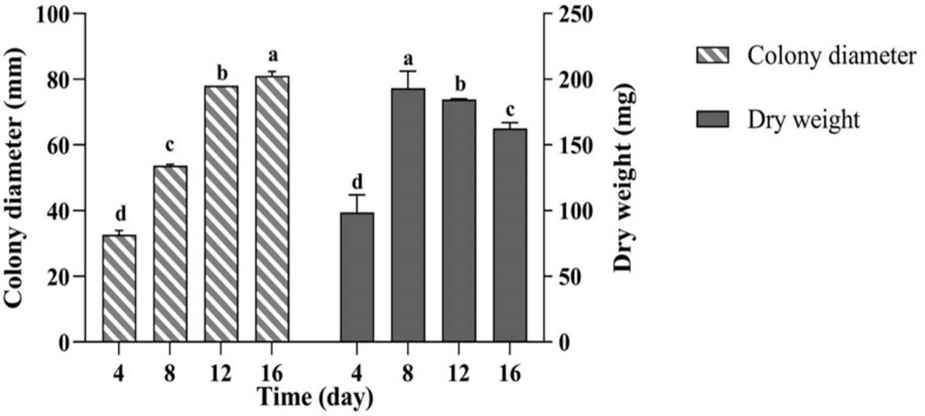

B.

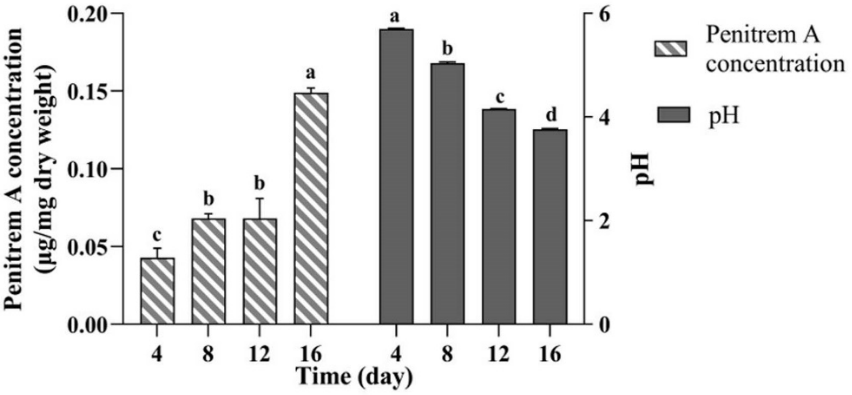

Figure S5. Growth of *P. crustosum* OM1 and its penitrem A production on PPAM (pH 6.5) agar plates at 22°C. (A) Colony diameter and mycelial dry weight, and (B) levels of penitrem A and pH of the media. The levels of penitrem A, dry weight of mycelia, colony diameter, and pH of the media were measured in triplicate. Data are expressed as the mean ± standard deviation. Different letters indicate statistically significant differences ($P<0.05$) in the same group.

Supplementary Tables

Table S1. Levels of penitrem A or patulin produced by fungal isolates and their scientific names identified by BLAST-based analysis.

Sample ID	Isolate or strain no. from NCBI [1]	Scientific name (BLASTn accession no.) *F. proliferatum* [2]	Patulin (μg/mL)	Penitrem A (μg/mL)
P1	Strain S6-Y-2-10	*Penicillium fellutanum* (KP216922.1)	ND [3]	ND
P2	Strain DQ-30	*Penicillium crustosum* (KY022755.1)	ND	0.62±0.01
P3	Isolate SRG2	*Aspergillus aculeatinus* (MK748310.1)	ND	- [4]
P4	Isolate RM101	*Penicillium citrinum* (KU613360.1)	ND	ND
P5	Isolate A35	*Aspergillus aculeatus* (MW186675.1)	ND	-
P6	Isolate ND44	*Aspergillus niger* (MG659638.1)	ND	-

[1]The isolate or strain names were retrieved from GenBank in National Center for Biotechnology Information (NCBI).

[2]BLASTn was run using ITS1-5.8S rDNA-ITS2 sequences. BLASTn indicates basic local alignment search tool for nucleotide, while ITS represents internal transcribed spacer.

[3]ND represents not detected.

[4]- indicates not tested.

Table S2. Percentages of penitrem A detected in 2 different parts of *P. crustosum* OM1 after 7 day culture in 3 different liquid media.

Culture condition	Medium	Penitrem A (%)	
		Mycelia	Culture filtrate
Static, 22 °C	mYES4	95	5
	PDB	100	ND [1]
	mMEB	100	ND

[1]ND indicates not detected.
